# *Gastrodia elata* Blume extract suppresses lipid accumulation in high-fat diet-fed rats: a biochemical and histopathological evaluation

**DOI:** 10.1186/s42826-025-00257-3

**Published:** 2025-10-16

**Authors:** Hyeon Jeong Na, Yeon Su Lee, Da Eun Jung, Ji Won Seo, Jeong Su Park, Jin Woo Hong, Jae-Ho Shin

**Affiliations:** Department of Biomedical Laboratory Science, Graduate School, Eulji University, Seongnam, 13156 Korea

**Keywords:** *Gastrodia elata* Blume, High-fat diet, Cholesterol, Liver, Rat

## Abstract

**Background:**

Excessive consumption of high-fat diet (HFD) can easily cause obesity, hyperlipidemia, and fatty liver disease. Persistent elevation of blood lipid levels increases the risk of cardiovascular disease, with hyperlipidemia being a well-established risk factor. *Gastrodia elata* Blume (GEB) is a perennial orchid plant that is known to have beneficial effects on obesity and blood circulation. This study aimed to evaluate the effects of GEB extract on improving blood lipids in the hyperlipidemia model induced by HFD in rats. Wistar rats (five-week-old) were divided into 3 groups: Control (CON) group, HFD induced (HF) group, and GEB treated (GEB) group.

**Results:**

The treatment of GEB extract reduced body weight gain, visceral fat, and epididymal fat weights. Serum total cholesterol (TC) levels were significantly lower in the GEB group than in the HF group. Alanine aminotransferase (ALT), aspartate aminotransferase (AST), and alkaline phosphatase (ALP) were reduced in the GEB group than in the HF group. Histopathological analysis of the liver showed that the GEB group alleviated structural damage to the liver by reducing lipid accumulation in hepatocytes compared with the HF group in hematoxylin and eosin (H&E) and Oil red O staining. The adipocyte diameter was smaller in the GEB group than in the HF group, and the atherosclerosis index (AI) was significantly lower in the GEB group compared to the HF group. Furthermore, the mechanism underlying these effects was elucidated by demonstrating that GEB extract decreased the expression of sterol regulatory element-binding protein 1 (SREBP-1), a key regulator of cholesterol synthesis in the liver.

**Conclusions:**

These results indicate that GEB extract has the effect of improving blood and liver lipid levels.

## Background

In recent years, high-fat foods such as fast food have become a predominant component of modern diet [1]. Additionally, the entrenched consumption of fast food and lack of exercise in modern lifestyles has been implicated in the rising prevalence of obesity and hypercholesterolemia [2]. Numerous studies have demonstrated that prolonged intake of a high-fat diet (HFD) leads to increase free fatty acid content and cause metabolic diseases such as hyperlipidemia, obesity, fatty liver, and cardiovascular disease [3, 4]. Cardiovascular disease remain the leading cause of mortality worldwide, and hyperlipidemia is recognized as one of the most critical risk factors influencing the prevalence and severity of cardiovascular disease [5–8].

It has been widely established that elevated levels of total cholesterol (TC) and low-density lipoprotein cholesterol (LDL) accelerate the onset and progression of cardiovascular disease [9]. Both LDL and high-density lipoprotein cholesterol (HDL) are types of lipoproteins, which are protein complexes responsible for transporting lipids in the bloodstream. LDL primarily facilitates the transport of cholesterol synthesized in the liver to peripheral tissues and cells, but elevated LDL levels are associated with adverse effects on cardiovascular health [10–12]. Moreover, excessive triglycerides resulting from hypercholesterolemia can be stored in adipose tissue and the liver [13].

Consequently, natural plant extracts with fewer adverse effects have garnered significant attention, and they hold potential for development as novel lipid-lowering agents [14, 15]. Various classes of natural products, such as polyphenols, flavonoids, saponins, and terpenoids, have been shown to suppress fat accumulation through diverse mechanisms of action [16, 17]. These include the inhibition of lipogenesis, promotion of lipid oxidation, and modulation of key transcription factors such as sterol regulatory element-binding protein 1 (SREBP-1), peroxisome proliferator-activated receptor alpha (PPARα), and 5′AMP-activated protein kinase (AMPK) pathways [18, 19]. *Gastrodia elata* Blume (GEB) is a perennial orchid plant that is mainly distributed in East Asia, including China, Korea, and Japan, and its rhizome is mostly used for medicinal purposes.

GEB has been reported to exhibit therapeutic effects on obesity, hypertension, circulatory improvement, and inflammatory diseases [20, 21]. GEB contains phenolic active components such as gastrodin that have been shown to lower blood pressure and alleviate cardiac hypertrophy [22]. However, limited studies have explored the effects of GEB extract on hyperlipidemia in animal models. Therefore, in this study, we investigated the effect and underlying mechanism of GEB extract in mitigating cholesterol accumulation in rats fed an HFD.

## Methods

### Preparation of GEB extract

**Fig. 1:**
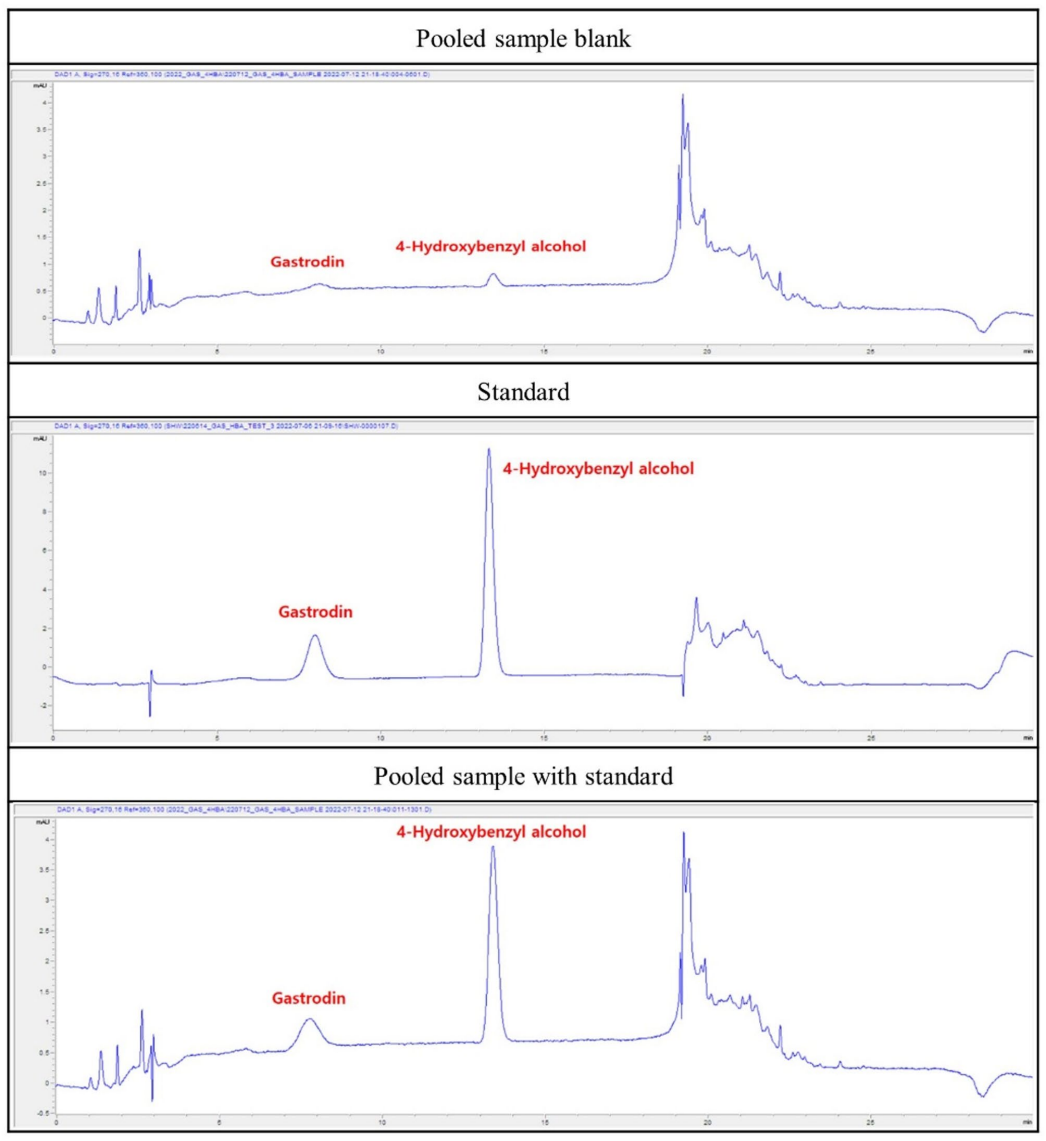
Chromatogram of gastrodin and 4-hydroxybenzyl alcohol analysis in GEB extract using HPLC-DAD (high performance liquid chromatograph diode-array detector)

The GEB was provided by the Rural Development Administration (Jeonju, Jeollabuk-do, Korea). The dried roots of GEB were subjected to hot air drying at 70℃ then the dried GEB was coarse-ground. Distilled water (DW) 10 times the weight (g) of coarse-ground GEB was added, extracted four times at 96 °C, and the first to fourth extracts were mixed. The mixed extract was lyophilized, then sterilized by radiation. For analysis of GEB extract, the following preprocessing steps were performed. 1 mL of GEB extract and 4 mL of 50% methanol were added and vortexed for 10 s. The mixed samples were subjected to ultrasonic extraction for 30 min. The ultrasonically extracted sample was centrifuged at 10,000 rpm for 10 min, and the supernatant was separated and filtered through a 0.45 μm syringe filter. Afterward, the preprocessed GEB extract was analyzed using high performance liquid chromatograph diode-array detector (HPLC-DAD). The analysis results showed that the peaks shown in Fig. 1 represent gastrodin and 4-hydroxybenzyl alcohol which are known to be active components of GEB. Then, the obtained GEB powder was mixed with DW to make fresh doses required for administration once a week at a concentration of 800 mg/mL and stored at -4℃ until use.

### Animals and experimental diets

Male Wistar rats aged 5 weeks (180 ~ 210 g) were purchased from Orient Bio (Gyeonggi-do, Korea). The animals had free access to food and tap water and were kept in controlled conditions (temperature 22 ± 2℃, humidity 50 ± 10%, and 12-h light/dark cycle). During the adaptation, the animals were fed a normal diet (ND) in the laboratory. To induce hyperlipidemia, animals were fed an HFD. The ND was a laboratory standard diet (Purina, Gyeonggi-do, Korea) which yielded a density of 2.97 kcal/g with 24.52% protein, 12.41% fat, and 63.07% carbohydrates. The HFD was purchased from Research Diet (#D12492, New Jersey, USA), which yielded of a density of 5.24 kcal/g with 20% protein, 60% fat, and 20% carbohydrates. The experimental procedure was approved by the Institutional Animal Care and Use Committee of Eulji University (EUIACUC 22−11).

### Experiment design

**Fig. 2:**
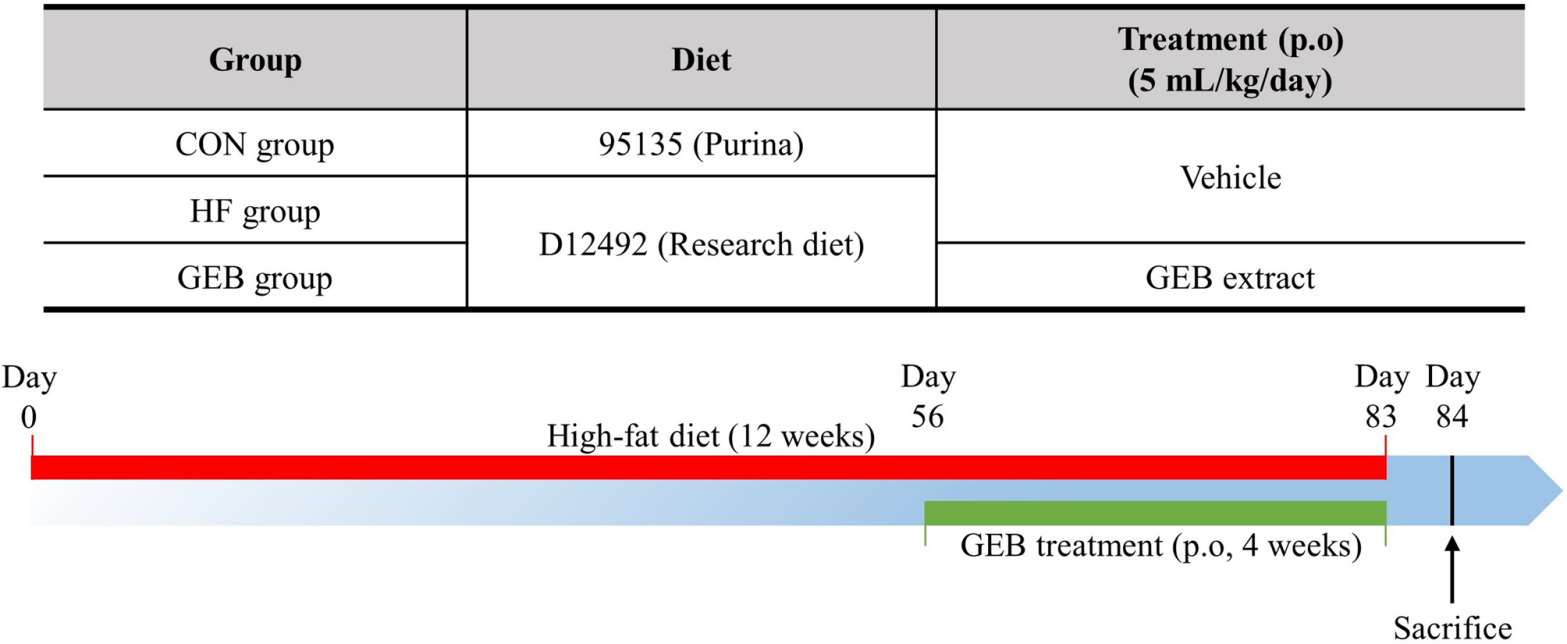
Experimental design and groups. The control (CON) group was fed a normal diet (ND), the high-fat (HF) group was fed a high-fat diet (HFD), and the GEB group received HFD along with Gastrodia elata Blume (GEB) extract treatment

After adaptation for 5 days, 27 male Wistar rats were divided into 3 groups (*n* = 7–10): the control (CON) group: rats fed the ND, the high-fat (HF) group: rats fed the HFD, and the GEB group: rats fed the GEB extract. The control group was fed an ND and the other groups were fed an HFD containing 60% kcal from fat for 12 weeks. Treatment was administered by oral gavage at 5 mL/kg of body weight, once a day for the last 4 weeks. The CON and HF groups were orally administered vehicle, whereas the GEB group was administered GEB extract. The body weight and food and water intake were measured twice per week. After the last treatment, the rats were allowed to be fasted overnight and anesthetized with isoflurane (Halocarbon, Georgia, USA) (Fig. 2). The blood samples were collected from the abdominal aorta of the rats into serum-separating tubes. The livers and fats were dissected and weighed promptly for histopathological analysis. The relative organ weight was calculated as the ratio of organ weight to the body weight and was expressed as a percentage. The left and half of the middle lobes of the liver, fat, and carotid artery samples were fixed in 10% neutral buffered formaldehyde for the hematoxylin and eosin (H&E) stain and immunohistochemistry (IHC). The other half of middle lobes of liver samples were fixed in 4% paraformaldehyde for the Oil red O stain. The right and caudate lobes of liver samples were frozen in liquid nitrogen and stored at -80 °C for molecular biological analysis.

### Biochemical measurements of lipid profile and liver function in serum

A total of blood samples were centrifuged at 3,000 rpm and 4 °C for 10 min, and the resulting serum was used for biological analyses and stored at -80 °C. To evaluate the lipid profile, the level of TC, HDL and LDL was measured, and the TC was measured using a cholesterol analysis kit purchased from Abcam (ab65390, Cambridge, MA, USA). The atherosclerosis index (AI) was calculated using the following formula: (TC-HDL)/HDL [23, 24]. To evaluate liver function, the serum levels of alanine aminotransferase (ALT), aspartate aminotransferase (AST), and alkaline phosphatase (ALP) were measured.

### Histopathological analysis

The fixed liver, fat, and carotid arteries were processed using an automated tissue processor and then embedded in paraffin wax. The liver and carotid artery were sectioned into 4 μm and epididymal fat tissues were sectioned into 8 μm using a microtome. The sections were deparaffinized with xylene and rehydrated by decreasing the ethanol concentrations, and then stained with H&E, and finally, slides were mounted using Permount (Fisher chemical, Seoul, Korea). Also, other liver tissues were fixed in 10%, 20%, and 30% sucrose aqueous then embedded in OCT compound and sectioned into 10 μm using a cryostat and then stained with Oil red O, and finally slides were mounted glycerol jelly (Sigma, St. Louis, MO, USA). All stained sections were subsequently examined using a light microscope (Olympus, Tokyo, Japan) with a digital camera attachment.

Hepatic steatosis was graded on a scale of 0 to 3 based on the percentage of the presence of fat within hepatocytes, as shown in Table 1: Grade 0 (minimum, hepatocytes containing fat < 5%), Grade 1 (mild, hepatocytes containing fat 5–33%), Grade 2 (moderate, hepatocytes containing fat 34–66%), and Grade 3 (severe, hepatocytes containing fat >66%) [25, 26]. The analysis was performed by 4 blinded investigators.

For histopathological analysis, the number of adipocytes was measured per unit area (200 ×), and the value was used for quantitative analysis as the average of two fields of view. The thickness of the carotid artery wall was measured and the average value was used for quantitative analysis. Two fields were randomly selected and measured perpendicular to the vessel wall from the innermost part of the tunica intima to the innermost part of the tunica media, and the values are the average of the 2 fields. The analyses were performed blindly by a single calibrated operator using ImageJ software.

**Table 1:** Hepatic steatosis grade

Grade	Hepatocyte containing fat
0 = minimum	< 5%
1 = mild	5–33%
2 = moderate	34–66%
3 = severe	> 66%

### Immunohistochemical analysis

For IHC, the liver tissues were sectioned (4 μm) on silane-coated slide glasses (Matsunami, Osaka, Japan). ABC-horseradish peroxidase kit (vector laboratories, Burlingame, CA, USA) was used for immunohistochemical analysis. All slides were dewaxed in xylene and rehydrated through a graded series of ethanol concentrations. Then the slides were pretreated with 1 X citrate buffer (pH 6.0) for 10 min in a microwave for antigen retrieval and cooled for 30 min at room temperature. The slides were covered with 1% H2O2 in methanol for 30 min to block the endogenous peroxidase. After washes with phosphate-buffered saline (PBS), the sections were blocked with 5% bovine serum albumin containing normal horse serum for 1 h 30 min at room temperature. Next, the primary antibody SREBP-1 (sterol regulatory element binding protein-1, diluted 1:400, Santa Cruz Biotechnology, Dallas, TX, USA) was incubated overnight at 4 °C. After incubation, the slides were rinsed in PBS and incubated with biotinylated horse anti-mouse IgG (Vector Laboratories, CA, USA), diluted in PBS for 1 h at room temperature. Next, the sections were rinsed in PBS and incubated with avidin-biotin complex reagent (Vector Laboratories, CA, USA) for 30 min at room temperature. After rinsing the slides with PBS, the slides were stained with 1 × 3,3-diaminobenzidine tetrahydrochloride (DAB, Sigma, St. Louis, MO, USA) diluted in the buffer for 4 min 40 s. After staining, slides were mounted using a Permount and examined using an Olympus microscope (Olympus Optical Co., Tokyo, Japan). For histopathological analysis, the positive cells were counted by analysis of IHC scores performed by 4 blinded investigators, and the number of positive cells was calculated as a percentage of the total number of cells.

### Western blot analysis

Liver tissues were homogenized in radioimmunoprecipitation assay buffer (RIPA) buffer. Proteins (30 µg) were separated on an 8% sodium dodecyl sulfate-polyacrylamide gel and transferred to polyvinylidene difluoride (PVDF) membranes for Western blot analysis. The membranes were incubated with 5% skim milk solution for 1 h 30 min and then incubated overnight at 4 °C with the following primary antibodies: anti-SREBP-1 (diluted 1:1000; Santa Cruz Biotechnology, Dallas, TX, USA) and β-actin (diluted 1:2000; Cell Signaling Tech, Beverly, MA, USA). After washing with Tris-buffered saline containing 0.1% Tween-20 (TBS-T), the membranes were incubated for 1 h with secondary antibody, anti-mouse IgG (diluted 1:2000; Cell Signaling Tech, Beverly, MA, USA). Protein bands were visualized by scanning using a Bio-Rad Chemidoc (Bio-Rad, Hercules, CA, USA) with the addition of Clarity Max ECL blotting substrates. The changes in protein expression were quantified using Imagelab software 6.1 (Bio-Rad, Hercules, CA, USA).

### Real-time quantitative polymerase chain reaction (RT-qPCR) analysis

SREBP-1 gene expression was determined by RT-qPCR. Total RNA was extracted from rat liver using TRIzol, and cDNA was synthesized from total RNA (1 µg) using a cDNA reverse transcription kit (TaKaRa, Tokyo, Japan). mRNA expression was then quantified through RT-qPCR using SYBR green master mix (A25741, Thermo Fisher Scientific, USA) and Quantstudio (Thermo Fisher Scientific, USA). The PCR conditions were as follows: initial denaturation at 95 °C for 10 min, followed by 40 cycles of denaturation at 95 °C for 30 s, annealing at 60 °C for 30 s, and extension at 72 °C for 30 s. A melting curve analysis was performed at the end of the amplification to verify specificity. The primer sequences of the genes are as follows: 5′-CATCAACAACCAAGACAGTG-3′ (forward primer, NM_001276707), and 5′-GAAGCAGGAGAAGAGAAGC-3′ (reverse primer, NM_001276707) for SREBP-1, 5′-AGGAGTACGATGAGTCCGGC-3′ (forward primer, NM_031144) and 5′-CGCAGCTCAGTAACAGTCCG-3′ (reverse primer, NM_031144) for β-actin. The cycle thresholds (Ct) were determined based on the intensity of SYBR green emission during the exponential phase. Ct data were normalized using β-actin, which was stably expressed across all experimental groups. Relative gene expression was calculated using the 2-ΔΔct method [27].

### Statistical analysis

Statistical comparisons were analyzed using a one-way analysis of variance, followed by the least significant difference post-hoc analysis with SPSS (version 28.0; SPSS Inc., Chicago, USA). Differences were considered statistically significant when the *p*-value was less than 0.05. All statistical analyses were expressed as mean ± SD.

## Results

### Effect of GEB extract on body, liver, and fat weights

The body and organ weight of rats in all groups are shown in Table 2. The mean initial body weights of the CON, HF, and GEB groups were similar. The body weight of rats in all groups gradually increased over 12 weeks. After a 4-week experimental administration period, weight gain was observed in the HF group compared to the CON group. On the other hand, the average body weight decreased in the GEB group compared to the HF group. Furthermore, food intake was reduced in the GEB group compared to the HF group. The weight of epididymal fat and visceral fat remarkably increased in the HF group compared to the CON group, while it remarkably decreased in the GEB group compared to the HF group. No significant differences were observed between relative liver weights in the GEB group compared to the HF group.

**Table 2:** Initial body weight, final body weight, relative fat and liver weights

	CON	HF	GEB
Initial body weight (at 8 week, g)	508.6 ± 28.6	602.2 ± 72.4 ^a^	619.0 ± 31.8 ^a^
Final body weight (at 12 week, g)	543.6 ± 39.4	645.2 ± 86.4 ^a^	623.6 ± 12.0
Epididymal fat/ body weight (%)	1.1 ± 0.3	2.7 ± 0.4 ^a^	2.0 ± 0.5 ^a, b^
Visceral fat/ body weight (%)	1.7 ± 0.3	4.0 ± 0.7 ^a^	3.2 ± 0.7 ^a, b^
Liver/ body weight (%)	2.5 ± 0.1	2.2 ± 0.1 ^a^	2.3 ± 0.2 ^a^

^a^ Compared with the CON group ( *p*  < 0.05); ^b^ Compared with the HF group ( *p*  < 0.05)

### Effect of GEB extract on the serum lipid profile and AI level

**Fig. 3:**
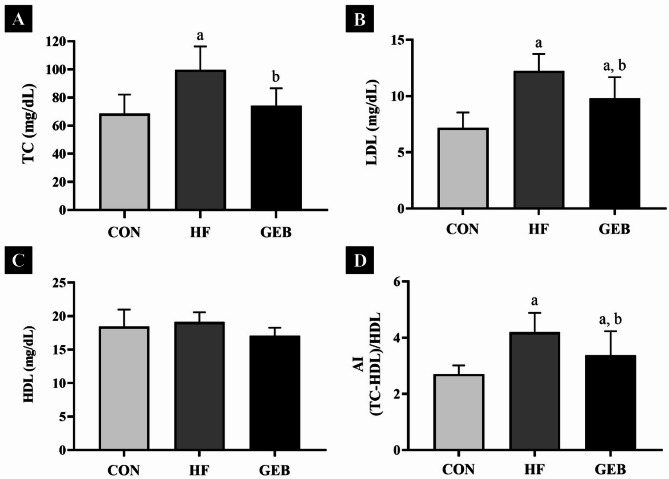
The levels of lipid profiles in serum on rats. (**A**) Serum TC (total cholesterol) level, (**B**) serum LDL (low-density lipoprotein cholesterol) level, (**C**) serum HDL (high-density lipoprotein cholesterol) level, and (**D**) AI (serum atherosclerosis index). Serum TC and LDL levels decreased in the GEB group compared to the HF group. The AI is the measure of the atherosclerotic lesion. AI value was markedly lower in the GEB group than in the HF group. Data are presented as mean ± SD (*n* = 7–10). ^a^ Compared with the CON group (*p* < 0.05); ^b^ Compared with the HF group (*p* < 0.05)

After 4 weeks of GEB extract treatment, serum levels are shown in Fig. 3. Serum TC levels were significantly higher in the HF group compared to the CON group and were significantly lower in the GEB group compared to the HF group (Fig. 3A). Serum LDL levels were significantly lower in the GEB group (Fig. 3B). No significant differences were observed between HDL levels in all groups (Fig. 3C). The AI value, a measure of atherosclerotic lesions, was significantly lower in the GEB group compared to the HF group (Fig. 3D).

### Effect of GEB extract on liver function

**Fig. 4:**
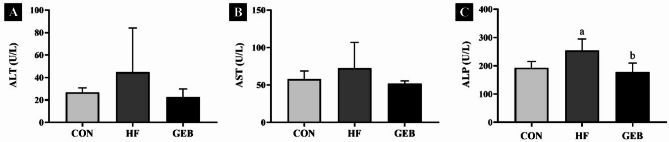
The levels of ALP, ALT, and AST in serum on rats. (**A**) ALT (alanine aminotransferase), (**B**) AST (aspartate aminotransferase), and (**C**) ALP (alkaline phosphatase). (**A**) The serum ALT, AST, and ALP levels were decreased in the GEB group compared to the HF group. Data are presented as mean ± SD (*n* = 7–10). ^a^ Compared with the CON group (*p* < 0.05); ^b^ Compared with the HF group (*p* < 0.05)

The ALT, AST, and ALP are important biomarkers of liver function (Fig. 4). ALT and AST levels in the GEB group showed a tendency to decrease compared with the HF group, and the levels fell in the normal range according to previous reports (Fig. 4) [28]. The ALP levels in the GEB group showed a significant decrease compared with the HF group.

### Histopathological changes in adipose tissue

**Fig. 5:**
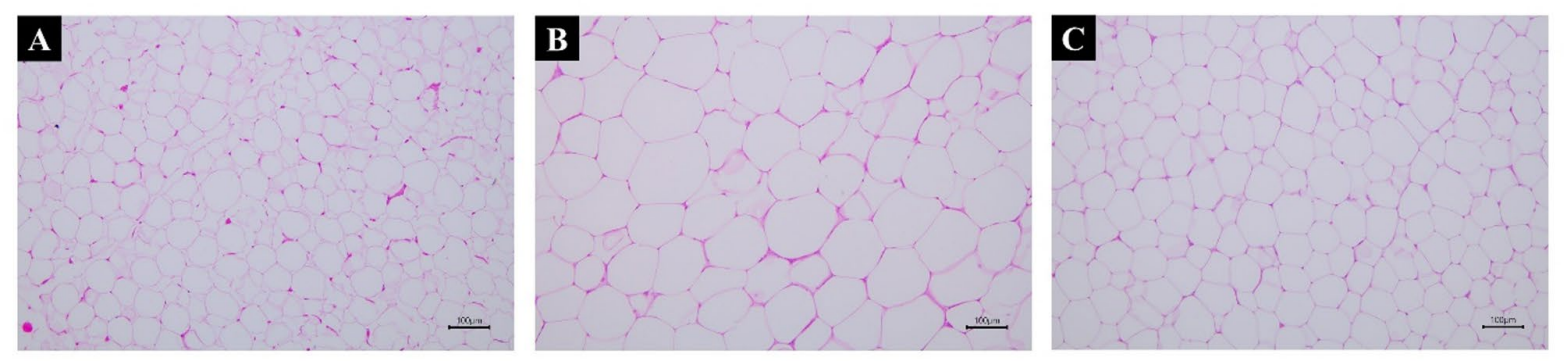
Histopathological changes in H&E-stained epididymal fat. (**A**) CON group, (**B**) HF group, (**C**) GEB group. To examine the accumulation of lipid droplets in epididymal fat in the CON, HF, and GEB groups. The increased size of adipocytes corresponds to the increased fat accumulation due to HFD. Scale bars in (**A**)-(**C**) 100 μm

The histopathological structure of the adipose tissue was analyzed through an H&E stain in epididymal fat (Fig. 5). The increased size of adipocytes corresponds to the increased fat accumulation due to an HFD. The diameter of epididymal adipocytes in the HF group was greater than the CON group and the epididymal adipocytes of the HF group distributed in an irregular manner. In the GEB group, the epididymal adipocytes became smaller than in the HF group. The morphology of epididymal adipocytes of the GEB group was similar to that of the CON group. When the number of adipocytes was measured within an area of ​​the same size, the number of adipocytes in the HF group decreased compared to the CON group, and the number of adipocytes in the GEB group increased compared to the HF group (Table 3).

**Table 3:** Changes in the number of adipocytes

Group	Number of adipocytes (200 ×, 1 mm^2^)
CON	57.2 ± 6.1
HF	30.2 ± 4.4 ^a^
GEB	44.6 ± 4.9 ^a, b^

^a^ Compared with the CON group ( *p*  < 0.05); ^b^ Compared with the HF group ( *p*  < 0.05)

### Histopathological changes in liver tissue

**Fig. 6:**
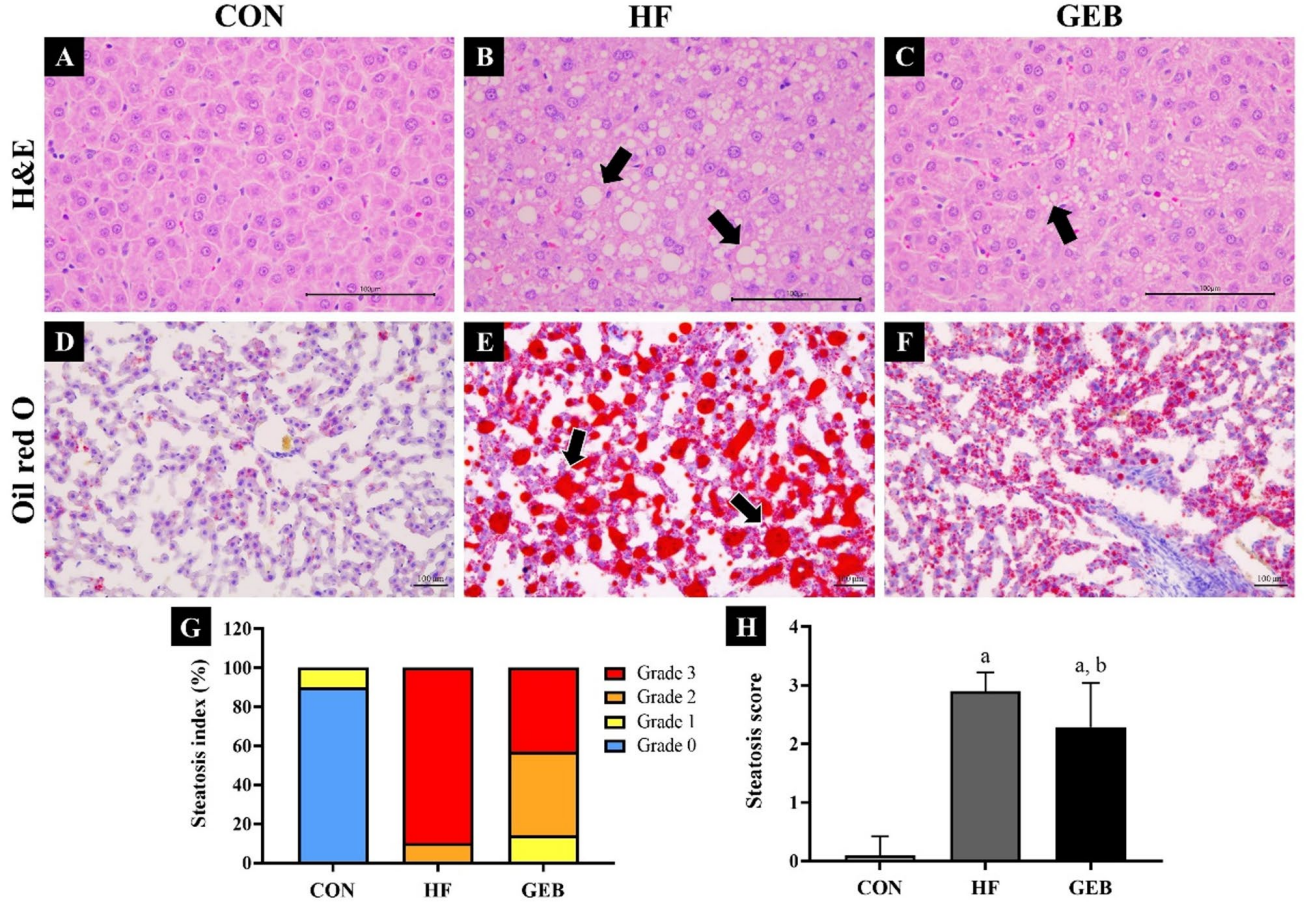
Histopathological changes in H&E and Oil red O-stained liver and hepatic steatosis scoring. (**A**)-(**C**) H&E, (**D-F**) Oil red O, (**A, D**) CON group, (B, E) HF group, (**C, F**) GEB group, and (**G**) Percentages of rats with each hepatic steatosis grade (0–3) for each group, (**H**) Mean of steatosis score. The result shows lipid deposition in hepatocytes. Compared to the CON group, the HF group showed loss of usual concentric arrangements of hepatocytes with fat vacuoles (arrow). On the other hand, in the GEB group, most of the cells had been restored to a level similar to that of the CON group and arranged normally. When the average grade for hepatic steatosis was analyzed, it was significantly decreased in the GEB group compared to the HF group. Scale bars in (**A**)-(**E**) 100 μm. Data are presented as mean ± SD (*n* = 7–10). ^a^ Compared with the CON group (*p* < 0.05); ^b^ Compared with the HF group (*p* < 0.05)

The histopathological structure of the liver was analyzed through H&E and Oil red O staining (Fig. 6). The tissue section of the CON group showed the normal structure of the liver. The hepatocytes had polygonal edges with clear cell borders, and their nuclei were round and clear, also located in the center of the cell with well-preserved cytoplasm (Fig. 6A). In comparison to the CON group, the HF group had macro-fat vacuoles large enough to distort the nuclei within hepatocytes, disrupting the sinusoid space and showing a loss of the typical concentric arrangement (Fig. 6B). On the other hand, in the GEB group, most of the cells had been restored and arranged normally, similar to the CON group (Fig. 6C). Oil red O-staining revealed that supplements with GEB extract exhibited less accumulation of hepatic lipid droplets, as compared with the HF group. The GEB group showed a similar trend to the CON group. The number and size of lipid droplets (arrows) were smaller in the GEB group compared to the HF group (Fig. 6E and F). These results were consistent with the H&E staining results, suggesting treatment with GEB extract reduced structural damage and lipid deposition in the liver.

Semi-quantitative analysis was performed on H&E stained slides, and the analysis was scored from 0 to 3 based on the fat contained in the observed hepatocytes according to Table 1. According to the semi-quantitative measurement results, the CON group had a high distribution in grade 0, while the HF group had a high distribution in grade 3. The grade distribution of the GEB group was evenly divided into grades 2 and 3 (Fig. 6G). Therefore, when the grades of each group were expressed as an average, the GEB group had a remarkably lower average grade than the HF group (Fig. 6H).

### Effect of GEB extract on SREBP-1 expression

**Fig. 7:**
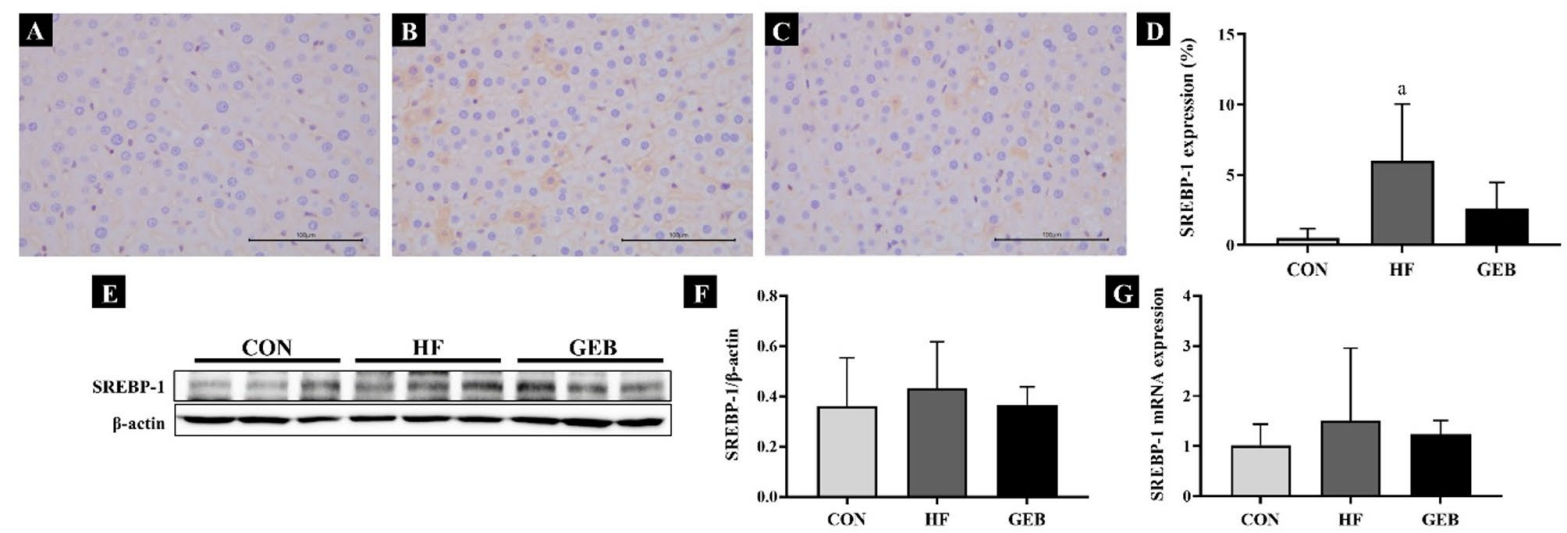
Effect of GEB extract on SREBP-1 (sterol regulatory element binding protein-1) expression in liver. (**A**) CON group, (**B**) HF group, (**C**) GEB group, (**D**) Percentage of SREBP-1 positive cell in IHC (immunohistochemistry), and (**E**), (**F**) protein expression of SREBP-1, (**G**) mRNA expression of SREBP-1 in the liver. Immunohistochemical staining of the liver for SREBP-1 in the CON group, HF group, and GEB group. The IHC signals were more intense and the number of positive cells was higher in the HF group than in the GEB group. Scale bars in (**A**)-(**C**) 100 μm. Expression of protein and mRNA of SREBP-1 was lower in the GEB group, compared with the HF group. Data are presented as mean ± SD (*n* = 7–10). ^a^ Compared with the CON group (*p* < 0.05)

To explore the mechanisms underlying the effects of GEB in the alleviation of lipid accumulation, the expression level of lipid synthesis-related proteins such as SREBP-1 was determined by IHC, Western blot, and RT-qPCR. The IHC signals were more intense and higher number of positive proteins in the liver of the rats in the HF group compared with the CON group (Fig. 7A and B). The GEB group showed mild signals and a lower number of positive proteins (Fig. 7C). The graphs (Fig. 7D) indicate the relative percentage of the expression of SREBP-1 in the liver tissue sections. According to the Western blot results (Fig. 7F), the protein expression of SREBP-1 in the HF group increased compared with the CON group, and the expression level of the protein in the GEB group decreased. Also, mRNA expression of SREBP-1 tended to decrease in the GEB group compared to the HF group (Fig. 7G), which is consistent with the IHC observations.

### Histopathological changes in carotid artery

**Fig. 8:**
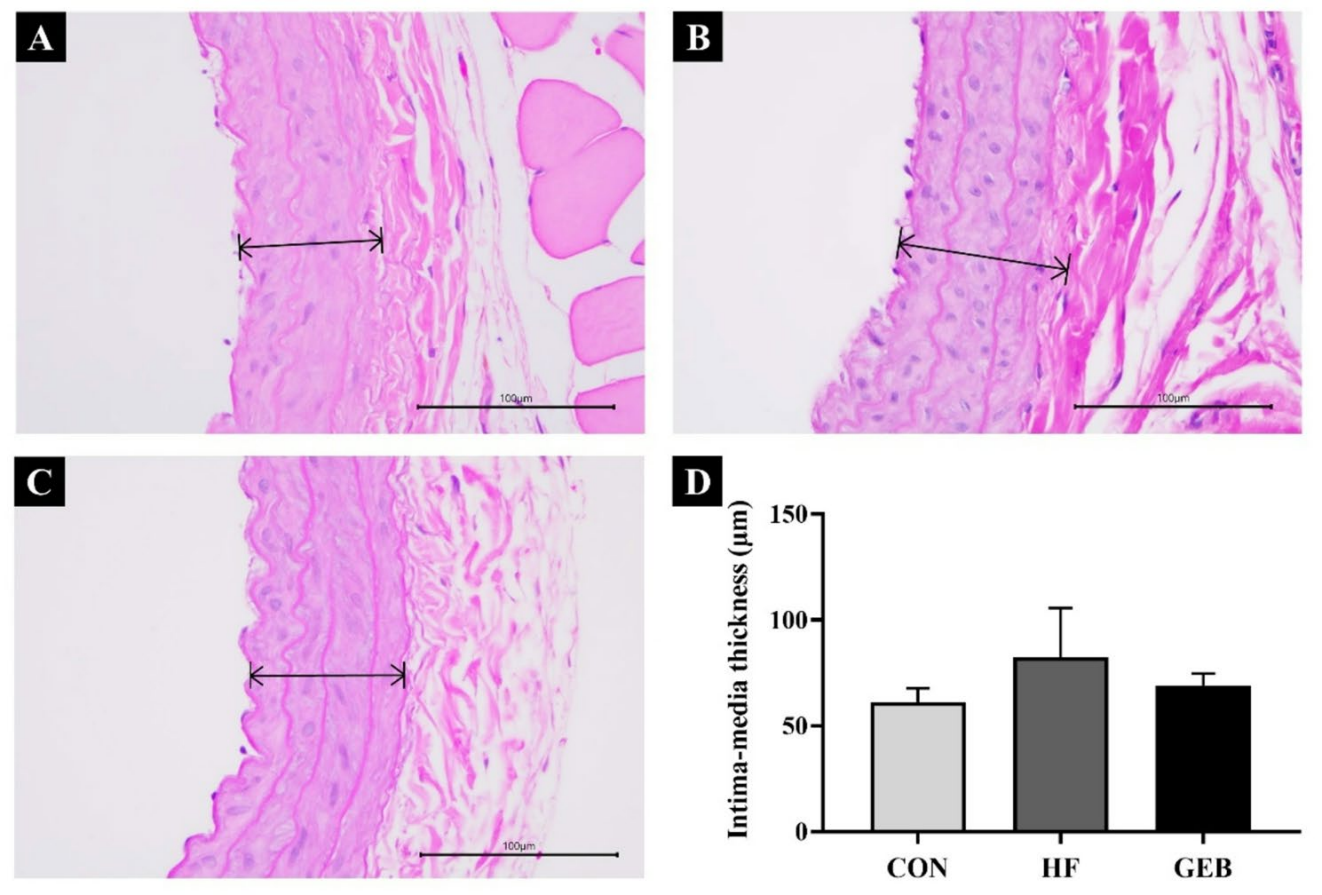
Histopathological changes and thickness measurement of the H&E-stained carotid artery. (**A**) CON group, (**B**) HF group, (**C**) GEB group, and (**D**) The thickness of the intima and media of the carotid artery wall (arrow). To examine carotid aortic histopathological changes in the CON group, HF group, and GEB group. The thickness of the intima and media of the carotid artery wall decreased in the GEB group compared to the HF group. Scale bars in (A)-(C) 100 μm. Data are presented as mean ± SD (*n* = 7–10)

Histopathological changes and differences in the carotid artery thickness were observed between the HFD-induced hypercholesterolemic rat groups (Fig. 8). The thickness of the carotid artery wall of the CON group was thin, and the endothelial cells were arranged in an orderly manner. In the HF group, the carotid artery wall was thicker, the endothelial cells were arranged disorderly, and several carotid artery wall ruptures were observed. In the GEB group, the arrangement of cell layers was closer to the CON group, and the carotid artery thickness was reduced compared to the HF group. As a result of measuring the thickness of the carotid artery from the innermost part of the tunica intima to the innermost part of the tunica media, the HF group showed increased thickness compared to the CON group. In comparison, the thickness of the carotid artery wall decreased in the GEB group (Fig. 8D).

## Discussion

Many studies have suggested that the continuous intake of an HFD is accompanied by weight gain and increased blood lipid levels leading to obesity, hypercholesterolemia, and insulin resistance [29–33]. Hypercholesterolemia is characterized by increased TC and LDL levels and decreased HDL levels, especially excessive LDL can accumulate in the blood vessel wall, which is a direct factor in the formation of atherosclerosis [9, 10]. Although there are currently drugs such as statins (e.g., atorvastatin, simvastatin, etc.) to treat hypercholesterolemia, statins have been reported to have many side effects including muscle weakness and muscle pain symptoms [34–36]. Over the past few years, clinical research has been conducted on the effects of plant extracts such as GEB on diseases including diabetes, hyperlipidemia, obesity, and cardiovascular disease [14, 15, 20, 35]. The natural properties of GEB may be a useful material for the treatment of hypercholesterolemia. Therefore, this study investigated the effect of GEB extract on alleviating hypercholesterolemia and protecting the liver in rats fed an HFD for 12 weeks.

In hypercholesterolemia, body weight change is one of the important pathological indicators. The weight of fat is a parameter that directly indicates the degree of obesity, and the main fats in the body include visceral fat and epididymal fat in rats. In this study, the weight gain of rats in the GEB group was found to be reduced compared to that of the HF group during the experimental period. These results are consistent with previous findings that GEB extract reduces body weight [37]. In addition, in the GEB extract administration group, the weight of epididymal fat and visceral fat was significantly reduced compared to the HF group. These results suggest that GEB extract alleviated fat accumulation and resulted in weight loss. Weight loss has important health implications, and weight loss of more than 5% can lead to improvements in blood biomarkers [38]. Moreover, body weight increased while dietary intake decreased in both the HF and GEB groups compared to the CON group, because HFD (5.24 kcal/mg) can efficiently increase body weight and body fat due to high-calorie intake even with a lower intake than ND (2.97 kcal/mg) [39, 40]. When the total calories consumed were calculated, the total calories consumed were similar in all three groups.

On the other hand, in this study, no significant difference was found in the liver weight of rats among all groups. These results may be because the metabolism that reduces fat in the blood or the intestinal absorption of lipids is more active than the metabolism that synthesizes and accumulates fat in the liver [41].

Several studies have reported that high long-term serum cholesterol levels increase the risk of developing atherosclerosis [29, 42, 43]. In particular, an increase in LDL levels is used as the most important pathological indicator in determining hypercholesterolemia. Increased reactive oxidized LDL in blood vessels can induce inflammatory responses and increase the risk of developing cardiovascular diseases such as atherosclerosis [7, 10, 11]. In this study, the results showed that serum TC and LDL levels were increased in the HF group confirming that hypercholesterolemia was induced in rats fed an HFD. In addition, the results showed that treatment of GEB extract significantly reduced TC and LDL levels in rats with HFD-induced hypercholesterolemia compared with the HF group. Consistent with our study, the cholesterol levels such as TC and LDL were confirmed to be significantly reduced in previous studies [41, 44].

Continuous intake of an HFD can cause cholesterol to be deposited on artery walls, causing them to thicken, which is also related to the AI, which is one of the important parameters in hypercholesterolemia studies [31, 45, 46]. AI is known to be a good indicator for predicting the risk of arteriosclerotic dyslipidemia in cardiovascular disease. The higher the arteriosclerosis index, the greater the risk of oxidative damage to these organs [47]. AI is calculated as the ratio of the difference between TC and HDL to HDL, AI was remarkably lower in the GEB group than in the HF group [23]. In addition, the histological changes of the carotid artery were observed through H&E staining, and the results showed that when GEB extract was administered, the cross-sectional thickness of the carotid artery decreased compared to the HF group, which was consistent with the decrease in the AI index. Therefore, the AI was remarkably reduced in GEB-treated rats, showing that GEB extract may be effective in preventing vascular dysfunction and atherosclerotic lesions, which may be directly related to the reduction in serum lipids. Consistent with our results, a previous studies found that adipose tissue weight and adipocyte size were significantly reduced in mice fed a high-fat diet [16, 48].

Administration of GEB extract prevented adipocyte hypertrophy in epididymal white fat. Previous studies have shown that continuous intake of an HFD causes excessive lipid accumulation in adipose tissue and increases adipocyte diameter [49, 50]. Histological analysis showed that the adipocyte diameter of the GEB group was reduced compared to the HF group (Fig. 5) and the number of adipocytes in one field (Table 3) was significantly increased in the GEB group compared to the HF group. This result is consistent with the tendency of decreased adipose tissue weight to decrease.

Serum ALT, AST, and ALP levels are biochemical indicators of hepatic damage [51, 52]. ALT, which is mainly found in the liver, is a biomarker of hepatocellular damage [53]. AST is an important biological marker for diagnosing amino acid metabolism [53]. Increased ALP activity indicates bile duct obstruction [53]. Fatty liver caused by intake of an HFD is accompanied by increases in ALT, AST, and ALP, and in this study, the ALP serum levels in the HF group were significantly higher than those in the CON group. The recent study showed that continuous intake of the GEB extract down-regulated ALT, and AST levels, which was also observed in this study [21]. Furthermore, levels of ALT and AST in the GEB group decreased close to those in the CON group, which is within the normal range reported in a previous study [28].

It has been known that continuous intake of an HFD causes fatty liver disease and increases liver weight, and long-term accumulation of lipids in the liver may cause liver metabolic dysfunction [26, 54, 55]. Hepatic steatosis is defined as the abnormal accumulation of fat within hepatocytes, with fat accumulation accounting for more than 5% of the liver weight or more than 5% of liver cells [56]. Fatty liver can be classified into alcoholic and non-alcoholic, and fatty liver caused by intake of an HFD corresponds to a non-alcoholic fatty liver. Non-alcoholic is defined as the presence of hepatic steatosis without hepatocyte damage in the form of hepatocyte swelling and is further subdivided into macrosteatosis and microsteatosis. Macrosteatosis is characterized by a single, large fat vacuole in the hepatocyte, with the nucleus moving to the periphery of the cell. In microsteatosis, the cytoplasm of the hepatocyte contains small lipid vesicles without nuclear movement [57]. Based on the results of observing the liver stained with H&E through an optical microscope, in the HF group, a larger number of fat droplets within hepatocytes, and macrosteatosis was observed, as in previous study [41, 44]. In contrast, the cell arrangement of the GEB group was similar to that of the CON group, microsteatosis, with a decrease in the number and size of lipid droplets, was mainly observed. Furthermore, Oil red O staining was performed to confirm the degree of lipid accumulation in the liver. As a result, the size and number of red-stained lipid vesicles decreased in the GEB group compared to the HF group. As a result of quantitative measurement, the HF group had a high distribution of almost severe grade 3, while the GEB group had a relatively moderate distribution of grade 2. Therefore, this suggests that 4 weeks of GEB extract treatment can prevent liver metabolic abnormalities by reducing fat accumulation and alleviating pathological structural damage to the liver.

To further confirm the mechanism by which GEB extract alleviates HFD-induced fat accumulation, SREBP-1 was evaluated. SREBP-1 is an important hepatic transcription factor that controls many genes involved in the metabolism of cholesterol and other lipids, regulating the rate of lipid synthesis and cholesterol synthesis [58, 59]. In previous studies on hepatic steatosis, these proteins were known to be increased when fat was accumulated in the liver, suggesting that increased cholesterol and fatty acid biosynthesis is a potential cause [60]. This study confirmed that GEB extract alleviated the increase in the levels of target proteins such as SREBP-1 in hypercholesterolemic rats. However, statistical significance was achieved only in the immunohistochemical results. This discrepancy may be attributed to the modest magnitude of change and relatively high inter-sample variability in the Western blot and RT-qPCR data. Nevertheless, these findings suggest that GEB may exert a lipid-lowering effect by downregulating SREBP-1-mediated lipogenesis, thereby contributing to the reduction of blood cholesterol levels and hepatic lipid accumulation. Further studies with greater statistical power are needed to confirm this mechanism.

This study showed that GEB reduced the expression of SREBP-1 which is a major transcription factor involved in lipid synthesis. However it did not examine whether this effect was related to upstream regulators such as AMPK, PPARα or liver X receptors (LXR). Previous studies have reported that AMPK activation can suppress lipogenesis driven by SREBP-1, and similar pathways may be involved in the action of GEB [61]. The exact mechanism by which GEB affects lipid metabolism could not be identified in this study and should be explored in future research. In addition, the current study employed a single dose of GEB extract, which was selected to initially confirm its efficacy in inhibiting fat accumulation in vivo. However, dose-response analyses and the identification of an optimal therapeutic dose suitable for clinical application require further investigation.

## Conclusions

**Fig. 9:**
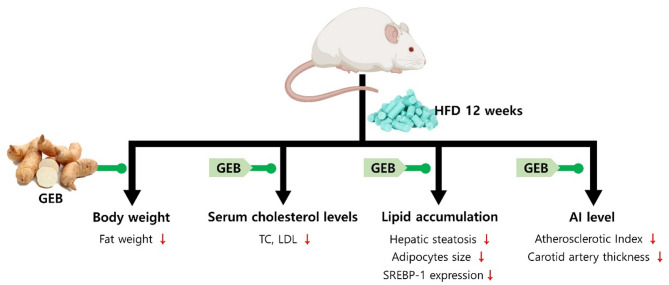
Effect of GEB extract on HFD-induced rat model. The treatment of GEB extract reduced lipid accumulation in blood and tissue of an HFD-induced rat model

In conclusion, administration of GEB extract for 4 weeks improved blood and tissue lipid levels in HFD-induced hypercholesterolemic rats (Fig. 9). It also alleviated structural changes in the liver, adipose tissue, and blood vessel walls by alleviating intravascular lipid accumulation. Taken together, these results suggest that GEB extract may be a potential candidate for preventing hypercholesterolemia. However, the mechanism by which GEB extract reduces blood lipid indices requires further studies.

## Data Availability

The datasets used and analyzed during the current study are available from the corresponding author on reasonable request.

## Abbreviations

AI: Atherosclerosis index
ALP: Alkaline phosphatase
ALT: Alanine aminotransferase
AMPK: 5′AMP-activated protein kinase
AST: Aspartate aminotransferase
CON: Control
DAB: 3,3-diaminobenzidine tetrahydrochloride
DW: Distilled water
GEB: *Gastrodia elata* blume
H&E: Hematoxylin & eosin
HDL: High-density lipoprotein cholesterol
HF: High-fat
HFD: High-fat diet
HPLC-DAD: High performance liquid chromatograph diode-array detector
IHC: Immunohistochemistry
LDL: Low-density lipoprotein cholesterol
LXR: Liver X receptors
ND: Normal diet
PBS: Phosphate-buffered saline
PPARα: Peroxisome proliferator-activated receptor alpha
PVDF: Polyvinylidene difluoride
RIPA: Radioimmunoprecipitation assay buffer
RT-qPCR: Real-time quantitative polymerase chain reaction
SREBP-1: Sterol regulatory element binding protein-1
TC: Total cholesterol
